# Prevalence, antibiotic profile, virulence determinants, ESBLs, and non-β-lactam encoding genes of MDR *Proteus* spp. isolated from infected dogs

**DOI:** 10.3389/fgene.2022.952689

**Published:** 2022-10-06

**Authors:** Reham M. El-Tarabili, Elsayyad M. Ahmed, Nada K. Alharbi, Maha A. Alharbi, Ahlam H. AlRokban, Doaa Naguib, Sadeq K. Alhag, Tamer Mohamed El Feky, Ahmed Ezzat Ahmed, Ahmed E. Mahmoud

**Affiliations:** ^1^ Department of Bacteriology, Immunology, and Mycology, Faculty of Veterinary Medicine, Suez Canal University, Ismailia, Egypt; ^2^ Department of Virology, Animal Health Research Institute (AHRI), Agriculture Research Center (ARC), Giza, Egypt; ^3^ Department of Biology, College of Science, Princess Nourah bint Abdulrahman University, Riyadh, Saudi Arabia; ^4^ Department of Hygiene and Zoonoses, Faculty of Veterinary Medicine, Mansoura University, Mansoura, Egypt; ^5^ Department of Biology, College of Science, King Khalid University, Abha, Saudi Arabia; ^6^ Animal Health Research Institute(AHRI), Mansoura laboratory Branch, Agriculture Research Center (ARC), Giza, Egypt; ^7^ Department of Theriogenology, Faculty of Veterinary Medicine, South Valley University, Qena, Egypt; ^8^ Department of Animal Medicine, Faculty of Veterinary Medicine, Suez Canal University, Ismailia, Egypt

**Keywords:** *antimicrobial resistance*, virulence genes, MDR, Proteus, dog, diarrhea, prevalence, Egypt

## Abstract

This study investigated the prevalence, antibiogram, virulence, extended-spectrum β-lactamases (ESBLs), and non-β-lactam encoding genes of *Proteus* species isolated from infected dogs in Ismailia province, Egypt. The study was conducted on 70 fecal swabs collected from dogs with diarrhea for bacteriological identification of *Proteus* spp*.* The positive isolates were evaluated for antibiotic susceptibility, molecular tests of virulence, ESBLs, and non-β-lactam encoding genes. Prevalence of *Proteus* spp. was 35.7% (25/70), including *Proteus mirabilis* (*n* = 23) and *Proteus vulgaris* (*n* = 2). The *Proteus* spp*.* prevalence revealed diversity, higher in males than females, in ages < 12 weeks. Investigation of antimicrobial resistance was found against penicillin and amoxicillin (100%), amoxicillin–clavulanic acid (32%), cephalosporins: cefotaxime and ceftazidime (36%), and monobactam: aztreonam (28%) as ESBLs, in addition to tetracycline (32%) and trimethoprim sulfamethoxazole (100%). The strains retrieved by PCR revealed *ure*C, *zap*A, and *rsb*A virulence genes with variant prevalence as 92%, 60%, and 52%, respectively. In addition, the recovered strains contained ESBL genes with a dramatic variable prevalence of 100%, 92%, 36%, and 32%, to *bla*_TEM_, *bla*_SHV_, *bla*_CTX-M_, and *bla*_OXA-1_, respectively, and non β-lactam encoding genes with a prevalence of 100%, 48%, 44%, 20%, and 12%, to *sul*1, *tet*A, *int*I1, *qnr*A, and *aad*A1. Moreover, 28% (7/25) of recovering strains were MDR (multidrug-resistant) up to four classes of antimicrobials, and 48% (12/25) of the examined strains were MDR up to three antimicrobial classes. In conclusion, to the best of our knowledge, our study could be the first report recording MDR *Proteus* spp. in dogs in Egypt.

## 1 Introduction

*Proteus* species are Gram-negative bacilli belonging to the Enterobacteriaceae family, which is widespread in both the human and animal gastrointestinal tracts, especially the members of the genus *Proteus* (Hegazy, 2016). It is the ultimate example of an opportunistic nosocomial pathogen (Jacobsen and Shirtliff, 2011). *P. mirabilis*, *P. vulgaris*, *P. penneri*, *P. hauseri*, *P. terrae*, and *P. cibarius*, with the unidentified genomic species, are included currently within the *Proteus* genus (Manos and Belas, 2006; Drzewiecka, 2016). *P. mirabilis* pathogens were isolated from humans, dogs, monkeys, pigs, sheep, goats, raccoons, cats, rodents, and other mammals. It is considered a part of the normal microbiota of the mammalian intestinal tract (Guentzel, 1996). In addition, *P*. *mirabilis* and *P*. *vulgaris* are widely disseminated in the environment, occurring in contaminated water, sewage, and soil. It is critical to decompose the animals’ organic substances (Rózalski et al., 1997).

The antimicrobial-resistant strains of *Proteus* from companion animals were recorded. Their infectious nature results from their resistance to antipathogenic chemicals, enabling them to still be intact in their environment (Hola et al., 2012). The developed antimicrobial resistance leads to failure in treatment associated with an adverse impact on the animal’s welfare. Bacterial pathogens with resistance properties against antimicrobials can have substantial public health consequences for human beings if isolates are transmitted from pets to their owners (Guardabassi et al., 2004; Lloyd, 2007; Algammal et al., 2020). The resistance to extended-spectrum β-lactam is mostly related to the production of extended-spectrum β-lactamases; ESBLs would lyse many β-lactam antimicrobial agents like penicillins, different generations of cephalosporins, and carbapenems (Magiorakos et al., 2012; Rubin and Pitout, 2014; Algammal et al., 2020; Algammal et al., 2021b). The chemical backbone of β-lactam antibiotics is the -lactam ring, which can be broken by these enzymes (Fadare and Okoh, 2021). The resistance of ESBL producers to many antibiotic classes is a major concern in clinical settings as it renders the treatment of individuals infected with these bacteria difficult and frequently impossible (Rawat and Nair, 2010). Resistance to sulfonamides usually develops primarily due to dihydropteroate synthase (DHPS) enzyme or their mutations carrying *fol*P gene involved in the biosynthesis of the nucleotide or *via* the development of alternative genes of DHPS, such as *sul*1, *sul*2, and *sul*3, showing low affinity to sulfonamides (Perreten and Boerlin, 2003; Yun et al., 2012). Sulfonamides are chemotherapeutic drugs that act as competitive inhibitors of the *fol*P gene-encoded DHPS. Widespread sulfonamide resistance is mostly caused by plasmid- and integron-borne *sul*1-3 genes that code encoding mutant DHPS enzymes that do not bind to sulfonamides (Sánchez-Osuna et al., 2019). Tetracycline-resistant genes are most commonly found on conjugated plasmids and transposons. On the other hand, other isolates have the necessary genes encoded on their chromosomes (Guillaume et al., 2000; Oppegaard et al., 2001). Efflux pumps, ribosome protection, and enzymatic deactivation are the critical mechanisms of tetracycline resistance acquired by acquiring *tet* genes. The *aad* A family of genes encodes aminoglycoside-3″-adenylyltransferase (AAD), which promote aminoglycosides (White and Rawlinson, 2001). The quinolone-resistant Enterobacteriaceae are growing worldwide. The *qnr* genes enhance resistance to nalidixic acid and reduce ciprofloxacin susceptibility (Crump et al., 2003). Class 1 integrons are frequently linked to multidrug resistance due to their ability to acquire or eliminate several antimicrobial resistance gene cassettes (Chen et al., 2017). Awareness of bacterial resistance against the antimicrobials, among the isolates of canine *Proteus* spp., is essential from a veterinary standpoint and an international public health perspective (Harada et al., 2014).

Currently, the pathogenic mechanism of *Proteus*-associated diarrhea remains unknown. It is necessary and useful to investigate the intestinal pathogenicity of *Proteus*, which can cause diarrhea. Few published studies have elucidated the pathogenic mechanism of *Proteus* diarrhea until now (Gong et al., 2019)*. Proteus* spp. invasiveness is related to several virulent factors, and virulence genes encoded in plasmids regulate these virulent factors (Manos and Belas, 2006). Swarming on solid surfaces is a prominent characteristic property of *Proteus* spp. Although several genes were linked with swarming, the *rsb*A gene is a necessary swarming phenomenon for swarming control (Rather, 2005). Urease is an essential biomarker in *Proteus* infection for developing bladder and kidney stones. Many urease genes, like *ure*C, are essential to the urease enzyme development process (Li and Mobley, 2002). The *zap* gene product is encoded by many genes essential for protease production, especially *zap*A for regulating the expression of IgA protease during differentiation between the swimmer and swarmer cells (Walker et al., 1999). Urease and protease are considered diagnostic and differential aspects that describe this genus member from other Enterobacteriaceae members (Ali and Yousif, 2015).

Since the risky infection of *Proteus* spp. in dogs in Egypt has not been comprehensively clarified yet, this study aimed to clarify the prevalence, antibiogram, virulence genes, and ESBLs and non-β-lactam encoding genes of different *Proteus* spp. isolates to explore the potential hazard of *Proteus* spp. in dogs.

## 2 Materials and methods

### 2.1 Ethical approval

The study was conducted in accordance with the applicable rules and regulations of the Animal Ethics Review Committee of Suez Canal University (AERC-SCU) in Egypt. The experimental methods and laboratory work followed bacterial isolation, biosafety, and quality standards. In this study, no animals were experimentally used; however, dog handling and samples were applied with the owners’ consent.

### 2.2 Study period and location

This study has been performed in a private small animal clinic in the Ismailia governorate, Egypt, during the period from April to September 2020.

### 2.3 Animals and clinical examination

Moreover, 70 diseased puppies of different breeds and sex aged between 3–6 months were examined in our study. All the admitted dogs were suffering from diarrhea. Clinical examinations of such cases were performed as described by Côté et al. (2015). Fecal swabs placed in peptone water (Oxoid, United Kingdom) were randomly and aseptically obtained from the diseased animals and transmitted directly to the laboratory for bacteriological examination.

### 2.4 Isolation of *Proteus* spp. for identification

The swabs were supplemented in peptone water (Oxoid, United Kingdom) for 24 h at 37°C, then enriched broth was passed on XLD (xylose lysine deoxycholate) agar, 5% sheep blood agar, MacConkey agar, and TSI agar (triple sugar iron) (Oxoid, United Kingdom) by using a sterile bacterial loop, and then incubated at 37°C for 24–48 h. Bacterial identification was performed based on the culture characteristics, swarming activity, hemolytic activity, morphological characteristics with Gram’s staining, and suspicious colonies of bacteria were biochemically identified by catalase, H_2_S generation, urease, methyl red, citrate utilization, oxidase, and lactose fermentation. As previously stated, the indole test was applied to differentiate between *P. mirabilis* and *P. vulgaris* (Quinn et al., 2002).

#### 2.4.1 Susceptibility of the antimicrobial agents

Antimicrobial susceptibility for *Proteus* spp. isolates was performed using the Mueller–Hinton agar (Oxoid, United Kingdom) involved in the disc diffusion method, in which 11 (*n* = 11) antimicrobial agents were used: amoxicillin (AMX; 10 μg), penicillin (P; 10 U), amoxicillin–clavulanic acid (AMC; 30 μg), ceftazidime (CAZ; 30 μg), cefotaxime (CTX; 30 μg), aztreonam (ATM; 30 μg), norfloxacin (NOR; 10 μg), nalidixic acid (NA; 30 μg), gentamicin (gen; 10 μg), tetracycline (TE; 10 μg), and sulfamethoxazole–trimethoprim (SXT; 25 μg) (Oxoid, United Kingdom). *E. coli*-ATCC 35218 was used as a quality control strain. The isolates were tested for ESBLs using cephalosporin indicators CAZ (30 μg) and CTX (30 μg) (Oxoid, United Kingdom). The diameter of inhibition zones was calculated as < 22 mm and < 27 mm, respectively (CLSI, 2017). The phenotype resistance patterns were classified as pan drug-resistant (PDR) and are resistant to all used antimicrobial agents; extensive drug-resistant (XDR) species is resistant to one agent in all but two antimicrobial classes, multidrug-resistant (MDR) species is resistant to one agent in three antimicrobial classes or drug-resistant (DR) species is resistant to less than one agent in three antimicrobial classes as stated by Magiorakos et al. (2012). The multiple antibiotic resistance index (MARI) was calculated following this mathematical equation: MARI = c/d. The “c’’ indicates the sum of the antimicrobial agents against which the bacteria exhibited resistance, while “d’’ indicates the total of those agents used.

#### 2.4.2 DNA extraction

Bacterial genomic DNA was extracted from the retrieved *Proteus* spp. isolates using the QIAamp DNA Mini Kit (QIAGEN Sciences Inc., Germantown, MD, United States/Cat. No. ID 51326) according to the manufacturer’s instruction manual.

#### 2.4.3 Virulence-determinant, extended-spectrum β-lactamases, and non-β-lactam encoding gene detection

Moreover, 25 positive isolates of DNA extracts were used for virulence-determinant genes by using the PCR targeting (*ure*C, *zap*A, and *rsb*A) genes, as previously conducted (Pathirana et al., 2018); ESBL genes (*bla*_TEM_, *bla*_CTX-M_, *bla*_SHV_, and *bla*_OXA-1_), as previously mentioned (Colom et al., 2003; Sharma et al., 2009); and non-β-lactam encoding genes for sulfonamides (*sul*1), tetracyclines (*tet*A), aminoglycosides (*aad*A1), quinolones (*qnr*A), and integrons (*int*I1), as previously illustrated (White et al., 2000; Randall et al., 2004; Robicsek et al., 2006; Ibekwe et al., 2011). All genes’ detection of PCR experiments in this study were carried out with a 50 μl total reaction volume as 5 μl of PCR buffer (10×), 1 μl 200 μM (from each dNTP of 10 mM dNTP mix), 4 μl of the bacterial template, 0.4 μl Taq DNA polymerase (5 U/L), 30 pmol per primer, and H_2_O was added up to 0.05 ml. The PCR cycling conditions and oligonucleotide primers (Thermo Fisher Scientific, United States) are presented in Table 1. Positive control strains, provided by the Animal Health Research Institute (AHRI), Dokki, Egypt, and the negative template control (NTC) were used in all assays. The amplified PCR products were visualized against a 100-pb DNA marker by agar gel electrophoresis on 1.5% agarose containing ethidium bromide 0.5 g/ml.

**TABLE 1:** Primer sequences and thermal profile used in PCR assay.

Target	Gene	Primer sequence	Product size (bp)	PCR thermal profile (35 cycles)			Reference
				Denaturation	Annealing	Extension	
Virulence	*ure*C	F- GTT​ATT​CGT​GAT​GGT​ATG​GG	317	94°C	56°C	72°C	Pathirana et al. (2018)
		R- ATA​AAG​GTG​GTT​ACG​CCA​GA		30 s	40 s	40 s	
	*rsb*A	F-TTGAAGGACGCGATCAGACC	467	94°C	58°C	72°C	
		R-ACTCTGCTGTCCTGTGGGTA		30 s	40 s	45 s	
	*zap*A	F-ACCGCAGGAAAACATATAGCCC	540	94°C	59°C	72°C	
		R-GCGACTATCTTCCGCATAATCA		30 s	40 s	45 s	
Tetracycline	t*et*A	F-GGTTCACTCGAACGACGTCA	576	94°C	55°C	72°C	Randall et al. (2004)
		R-CTGTCCGACAAGTTGCATGA		30 s	40 s	45 s	
Sulfonamide	*sul*1	F-CGGCGTGGGCTACCTGAACG	433	94°C	54°C	72°C	Ibekwe et al. (2011)
		R-GCCGATCGCGTGAAGTTCCG		30 s	40 s	45 s	
Aminoglycoside	*aadA1*	F: TAT​CAG​AGG​TAG​TTG​GCG​TCA​T	484	94°C	50–54°C	72°C	Randall et al. (2004)
		R: GTT​CCA​TAG​CGT​TAA​GGT​TTC​ATT		30 s	40 s	45 s	
Quinolone	*qnrA*	F: ATT​TCT​CAC​GCC​AGG​ATT​TG	516	94°C	55°C	72°C	Robicsek et al. (2006)
		R: GAT​CGG​CAA​AGG​TTA​GGT​CA		30 s	40 s	45 s	
Integron 1		F: TGCGGGTYAARGATBTKGATTT	491	94°C	54°C	72°C	White et al. (2000)
		R: CARCACATGCGTRTARAT		30 s	40 s	45 s	
ESBL	*blaC*_TX-M_	F-ATGTGCAGYACCAGTAARGTKATGGC	593	94°C	54°C	72°C	Archambault et al. (2006)
		R-TGGGTRAARTARGTSACCAGAAYCAGCGG		30 s	40 s	45 s	
	*bla*_OXA-1_	F-ATATCTCTACTGTTGCATCTCC	619	94°C	54°C	72°C	Colom et al. (2003)
		R-AAACCCTTCAAACCATCC		30 s	40 s	45 s	
	*bla*_TEM_	F-ATCAGCAATAAACCAGC	516	94°C	54°C	72°C	
		R-CCCCGAAGAACGTTTTC		30 s	40 s	45 s	
	*bla*_SHV_	F-AGGATTGACTGCCTTTTTG	392	94°C	54°C	72°C	
		R-ATTTGCTGATTTCGCTCG		30 s	40 s	40 s	

#### 2.4.4 Statistical data analyses

Data were analyzed by the chi-squared test according to Feldman et al. (2008) by R software (version 4.0.2, www.r-project.org), and the differences between groups were considered significant at *p* < 0.05. Visualization of *Proteus* isolates according to the virulence and resistance genes with a heatmap supported by hierarchical clustering (dendrogram) was performed according to Kolde (2019), and cor and ggcorrplot packages were used to identify the correlation among the various antimicrobial agents. Fisher’s exact test and odds ratio [confidence interval (CI) = 0.95%] were determined among sex, age, and breed of examined dogs.

## 3 Results

### 3.1 Clinical manifestation presentation in dogs

All examined dogs showed an increased body temperature higher than or equal to 39.5°C, lethargy, anorexia, depression, dehydration, and foul-smelling watery bloody diarrhea. The record increase in body temperature progressed to subnormal later in the late stage of disease; also, the intensity of dehydration increased in parallel to diarrhea.

### 3.2 Phenotype and prevalence of the *Proteus* spp. in examined samples

Moreover, 25 *Proteus* spp. isolates were isolated from 70 fecal swabs obtained from sick dogs exhibiting diarrhea, depending on the phenotypic characteristics of colonies, microscopic morphology, and biochemical markers. On XLD, the colonies were red with a black center but appeared pale (non-lactose fermenter) on MacConkey agar, black on TSI (H_2_S producer), and hemolytic on the blood agar with clearly detected swarming activity. The biochemical tests of those isolates were positive for catalase, H_2_S generation, urease, methyl red, and citrate utilization. In contrast, a negative response to oxidase, lactose fermentation, and indole was detected, but the Voges–Proskauer tests were positive for *Proteus* spp. The indole test was used to differentiate between *P. vulgaris* and *P. mirabilis* as it was positive for *P. vulgaris* and negative for *P. mirabilis*. The overall prevalence of *Proteus* spp. was 35.7% (25/70) as 92% (23/25) vs. 8% (2/25) for *P. mirabilis* and *P. vulgaris*, respectively. In addition, 25.7% of the examined diseased animals (*n* = 18) are infected with other bacterial pathogens, including, *E. coli* (13/70, 18.6%), *Klebsiella pneumoniae* (4/70, 5.7%), and *Pseudomonas aeruginosa* (1/70, 1.4%).

### 3.3 Risk factors for *Proteus* spp. prevalence in dogs

**FIGURE 1:**
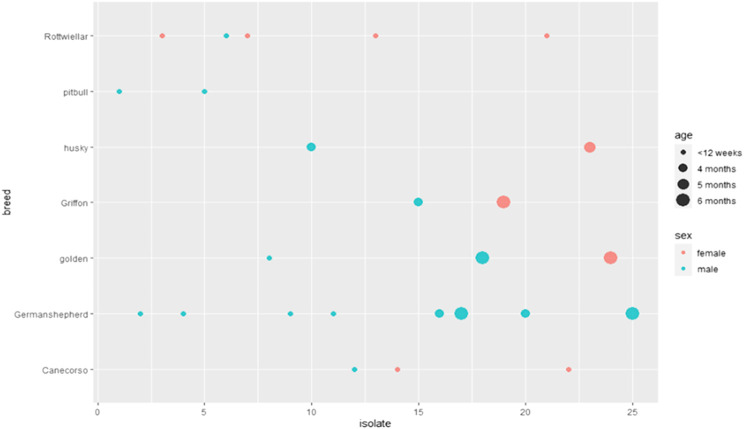
Prevalence of *Proteus* spp. isolated from dogs from clinics.

Prevalence of *Proteus* spp. varied with sex, age, and breed of examined dogs. It was higher in males, 64% (16/25) than in females 36% (9/25). It showed 60% (15/25) in dogs less than 12 weeks old rather than those of 4-, 5-, and 6 months old; 16% (4/25), 4% (1/25), and 20% (5/25), respectively. Concerning breeds, the prevalence rates showed 32% (8/25) in German Shepherd, 20% (5/25) in Rottweiler, 12% (3/25) in both Golden Retriever and 12% (3/25) Cane Corso, 8% (2/25) in each of Husky, 8% (2/25) Griffon, and 8% (2/25) Pitbull. Statistically, no significant difference was detected among the retrieved isolates according to age, gender, and breed, as exhibited in Table 2 and Figure 1.

**TABLE 2:** Odds ratio of *Proteus* isolates among age, sex, and breed.

	OR	95% CI	Chi-squared	*p*-value
Age	1.193489	0.7001873 2.108472	4.4707	0.2149
Sex	1.5000000	0.4368678 5.420620	0.10686	0.7437
Breed	0.9386627	0.7519946 1.165913	9.0488	0.3382

### 3.4 Antibiotic-resistant phenotypes of retrieved strains

**FIGURE 2:**
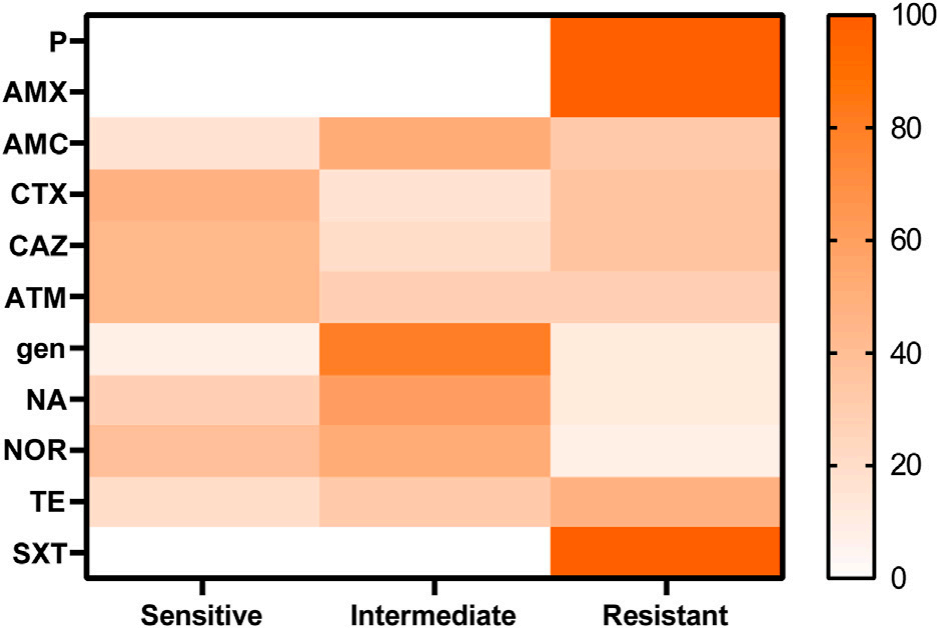
Antibiotic-resistant phenotypes of the recovered isolates of *Proteus*.

Susceptibility of the antimicrobial agents of the recovered *Proteus* spp. isolates revealed resistance to penicillins: penicillin and amoxicillin (100%), amoxicillin and clavulanic acid (32%); cephalosporins: cefotaxime and ceftazidime (36%); and monobactam: aztreonam (28%) as extended-spectrum β-lactamases, tetracycline (32%), and sulfonamides: trimethoprim sulfamethoxazole (100%), while both gentamicin and nalidixic acid showed low resistance (12%), as shown in Table 3; Figure 2. A significant difference was detected in the resistance of recovered isolates to the antibiotics tested (*p* < 0.05).

**TABLE 3:** Antibiotic-resistant phenotypes of the recovered isolates of *Proteus*.

Antibiotic class		Tested antibiotic	Interpretation					
			Sensitive		Intermediate		Resistance	
			*N*	%	*N*	%	*N*	%
β-Lactam-β-lactamase inhibitor combinations	Penicillins	Penicillin	—	—	—	—	25	100
		Amoxicillin	—	—	—	—	25	100
		Amoxicillin–clavulanic acid	4	16	13	52	8	32
	Cephalosporins	Cefotaxime	12	48	4	16	9	36
		Ceftazidime	11	44	5	20	9	36
	Monobactam	Aztreonam	11	44	7	28	7	28
Aminoglycosides	Aminoglycosides	Gentamicin	2	8	20	80	3	12
Quinolones	Quinolones	Nalidixic acid	7	28	15	60	3	12
	Fluoroquinolones	Norfloxacin	10	40	13	52	2	8
Tetracycline	Tetracycline	Tetracycline	5	20	8	32	12	48
Sulfonamide	Sulfonamides	Trimethoprim–Sulfamethoxazole	—	—	—	—	25	100
Chi-square*—p*-value			40.903		59.553		71.031	
			*p* < 0.0001		*p* < 0.0001		*p* < 0.0001	

### 3.5 Antimicrobial agents’ correlation

**FIGURE 3:**
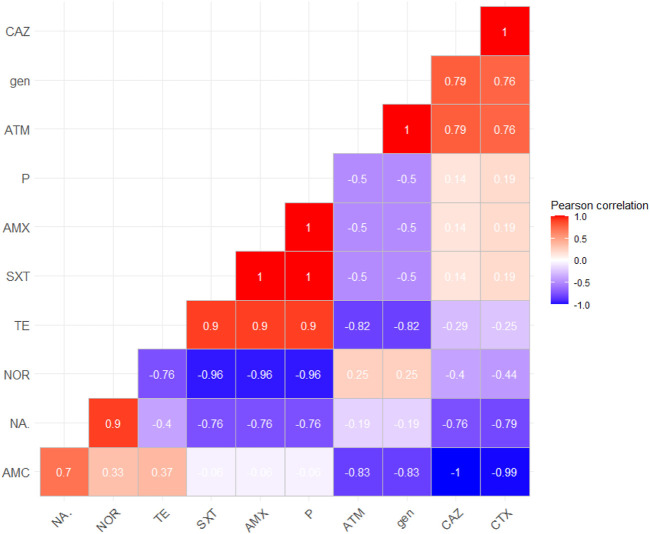
Correlation between antimicrobial agents; the red color detects the positive correlation, the darker red denotes a stronger positive correlation coefficient (0.5:1), the blue color detects negative correlation, and the darker blue detects a stronger negative correlation coefficient (−0.5:−1). AMX, amoxicillin; P, penicillin; AMC, amoxicillin–clavulanic acid; CAZ, ceftazidime; CTX, cefotaxime; ATM, aztreonam; NOR, norfloxacin; NA, nalidixic acid; gen, gentamicin; TE, tetracycline; SXT, sulfamethoxazole–trimethoprim.

Positive correlations were detected between amoxicillin, penicillin, and trimethoprim–sulfamethoxazole, between cefotaxime and ceftazidime, and also between cefotaxime, ceftazidime, and amoxicillin and clavulanic acid that confirm the relation between ESBL agents and finally between nalidixic acid, norfloxacin, and gentamicin, as illustrated in Figure 3.

### 3.6 Distribution of the virulence-determinant factors among the examined isolates

**FIGURE 4:**
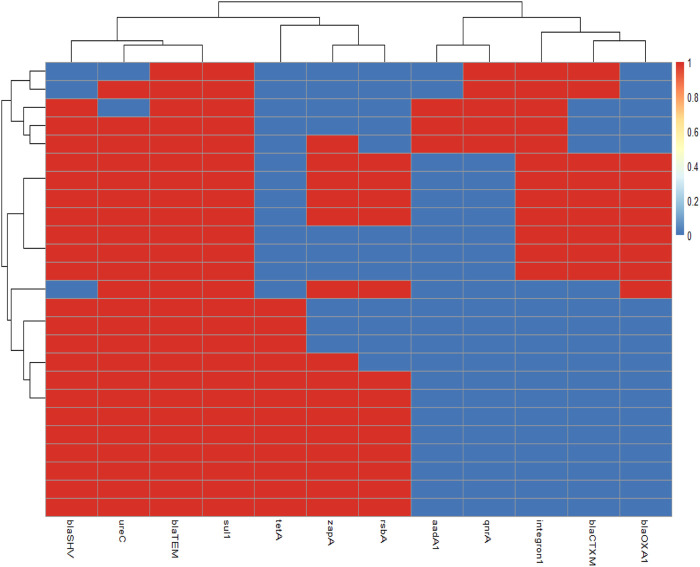
Overall distribution of virulence genes and antibiotic resistance profiles in *Proteus* isolates is represented by a heatmap supported by hierarchical clustering (dendrogram). The red color shows the presence of a gene, whereas the blue color shows the absence of a gene.

PCR confirmed that those examined strains harbored three virulence genes *ure*C, *zap*A, and *rsb*A with variable prevalence as 92% (23/25), 60% (15/25), and 52% (13/25), respectively (Table 4; Figure 4). No significant difference was detected in prevalence of the virulence-determinant genes among the different *Proteus* spp. (*p* < 0.05).

**TABLE 4:** Virulence-determinant gene prevalence of recovered isolates of *Proteus*.

Gene type		*N*	%	Chi-square—*p*-value
Virulence-determinant genes	*ure*C	23	92	3.2941—0.1926^NS^
	*zapA*	15	60	
	*rsbA*	12	52	

### 3.7 Detection of potential extended-spectrum β-lactamase genes

Phenotypically, ESBLs showed 32% (9/25) of the recovered isolates. However, the genes of antimicrobial resistance, *bla*_TEM_, *bla*_SHV_, *bla*_CTX-M_, and *bla*_OXA-1_, as detected in the isolates by PCR with specific predicted bands, were successfully amplified, proving that the examined strains contained ESBL genes with a dramatic variable prevalence as 100% (25/25), 92% (22/25), 36% (9/25), and 32% (8/25), respectively (Table 5; Figure 4). Significant differences among the ESBL gene prevalence of the recovered strains were detected (*p* < 0.05).

**TABLE 5:** Prevalence of ESBL and non-ESBL genes of recovered isolates of *Proteus*.

	Gene type	*N*	%	Chi-square—*p*-value
ESBL resistance genes	*bla*_TEM_	25	100	14.375—0.002437
	*bla*_*SHV*_	22	92	
	*bla*_CTX-M_	9	36	
	*bla*_OXA-1_	8	32	
Non-β-lactam encoding genes	*sul1*	25	100	25.803
	*tetA*	12	48	*p* < 0.001
	*intI1*	12	48	
	*qnrA*	5	20	
	*aadA1*	3	12	

### 3.8 Detection of integron class 1 and non-extended-spectrum β-lactamase genes

The integron (*int*I1) was detected at a prevalence of 44% (11/25), and the sulfonamide antimicrobial resistance gene (*sul*1) detected in *Proteus* spp. isolates showed a prevalence of 100% (25/25), while the tetracycline resistance gene (*tet*A) was detected at a prevalence of 48% (12/25), the quinolone resistance gene (*qnr*A) was detected at a prevalence of 20% (5/25), and the aminoglycoside resistance gene (*aad*A1) was detected in a prevalence of 12% (3/25) (Table 5; Figure 4). A significant difference was recorded in the sulfonamide or tetracycline gene prevalence in the recovered strains.

### 3.9 The relation between the phenotypic- and genotypic-resistant patterns among *Proteus* spp. isolates

The present findings found that 28% (7/25) of recovered isolates were multidrug-resistant to five antimicrobial families, penicillins: amoxicillin and ampicillin, cephalosporins: cefotaxime and ceftazidime, β-Lactam-β-lactamase inhibitor combination: amoxicillin–clavulanic acid, sulfonamides: trimethoprim–sulfamethoxazole, and tetracyclines: tetracycline, in response to the following resistance genes *bla*_TEM_, *bla*_SHV_, *bla*_OXA-1_, *bla*_CTX-M_, and *sul*1. In addition, 48% (12/25) of those isolates were resistant to three antimicrobial families; penicillins: amoxicillin and ampicillin, sulfonamides: trimethoprim–sulfamethoxazole, and also tetracyclines: doxycycline, in response to *bla*_TEM_, *bla*_SHV_ *, tet*A, and *sul*1 genes. Furthermore, 12% (3/25) of the tested strains revealed MDR to four antimicrobial families aminoglycoside: gentamicin, penicillins: amoxicillin and ampicillin, quinolones: nalidixic acid, and sulfonamides: trimethoprim–sulfamethoxazole, with the resistant genes such as *bla*_TEM_, *bla*_SHV_, and *sul*1 (Table 6; Figure 4). Multiple antibiotic resistance index (MARI) values in this study (≥0.2) exhibited resistance patterns of recovered isolates obtained from a high-risk contamination level (Table 6).

**TABLE 6:** Distribution of phenotypic resistance antibiotics and antimicrobial resistance genes among the examined isolates.

Isolate No.	%	Resistance type	Multidrug resistance phenotype	ESBLs and non-β-lactam encoding genes	MARI
12	48	MDR	Penicillins: AMX and P	*bla*_TEM_, *bla*_*SHV*_, *tetA*, and *sul1*	0.36
			Sulfonamides: SXT		
			Tetracyclines: TE		
7	28	MDR	Penicillins: AMX and P	*IntI1*, *bla*_TEM_, *bla*_*SHV*_, *bla*_*OXA-1*_, *bla*_CTX-M_, and *sul1*	0.63
			β-Lactam-β-lactamase inhibitor combination: AMC		
			Cephalosporins: CTX and CAZ		
			Sulfonamides: SXT		
			Monobactam: ATM		
3	12	MDR	Penicillins: AMX and P	*IntI1*, *bla*_TEM,_ *bla*_SHV_, *aadA1*, *qnrA*, and *sul1*	0.45
			Sulfonamides: SXT		
			Aminoglycosides: gen		
			Quinolones: NA		
2	8	MDR	Penicillins: AMX and P	*IntI1*, *bla*_TEM_, *bla*_*CTX-M*_, *qnr*A, and *sul1*	0.54
			Cephalosporins: CTX and CAZ		
			Sulfonamides: SXT		
			Fluoroquinolones: NOR		
1	4	DR	Penicillins: AMX and P	*bla*_TEM_, *bla*_*OXA-1*_, and *sul1*	0.36
			β-Lactam-β-lactamase inhibitor combination: AMC		
			Sulfonamides: SXT		

AMX, amoxicillin; P, penicillin; AMC, amoxicillin–clavulanic acid; CAZ, ceftazidime; CTX, cefotaxime; ATM, aztreonam; NOR, norfloxacin; NA, nalidixic acid; gen, gentamicin; TE, tetracycline; SXT, sulfamethoxazole–trimethoprim.

## 4 Discussion

Studies in human medicine investigated the microbial activities of *Proteus* (Chen et al., 2012; Decôme et al., 2020) but still need to be unveiled in veterinary medicine (Wong et al., 2015). The present study is a novel investigation of prevalence, PCR-based virulence, antimicrobial drug resistance, and ESBL genes contributing to pathogenicity of the *Proteus* spp. isolated from canine fecal samples in Egypt. Few studies are concerning the emergency of the *Proteus* spp. in canines. In this study, the overall prevalence of *Proteus* spp. in feces of clinically diseased dogs was 37.5%. Two species were identified, with the most prevalent species being *P. mirabilis*, followed by *P. vulgaris*. This is less than the 13.6% infection rate of *Proteus* spp. in dogs with urinary tract diseases in Thailand (Amphaiphan et al., 2021). In another study, *P. mirabilis* was detected in 22.7% of dogs’ urinary samples in the United Kingdom (Fonseca et al., 2021). In addition, domestic dogs in Nigeria had a 28% prevalence of *Proteus* spp. in their feces (Obioma et al., 2020). The phenotypic attributes of recovered *Proteus* spp*.* strains were unambiguous and revealed a high degree of agreement between them: red-colored and centrally black colonies on the XLD agar, pale colonies (non-lactose fermenter) on the MacConkey agar, black colonies on TSI agar, and characteristic swarming activity of the colonies (characteristics for *Proteus* spp.). Catalase, H_2_S, urease, methyl red, and citrate utilization are all positive for the recovered isolates. However, oxidase, lactose fermentation, Voges–Proskauer, and indole tests are all negative, except for *P. vulgaris*, which is positive for indole. Our results agreed with findings reported by Reich et al. (2013) and Lei et al. (2016). The conventional biochemical tests are necessary for the differentiation of *Proteus* and *Morganella*, and some tests are used for differentiation between them as swarming is characteristic for *Proteus* spp. and also H_2_S (O’Hara et al., 2000).

According to our findings, the recovered isolates showed resistance to penicillins, cephalosporins, monobactams (extended-spectrum β-lactamases), sulfonamides, and tetracycline, while both gentamicin and nalidixic acid showed low resistance. MARI values were ≥ 0.2, so it exhibited multiple resistance patterns, denoting that the recovered isolates were taken from high-risk contamination and that *P. mirabilis* from dogs’ isolates has high MARI (Zhang et al., 2018). Small animal bacterial infections were frequently treated with broad-spectrum antimicrobials such as penicillins and tetracyclines. The antimicrobials’ misuse in small animals and the ability of *P*. *mirabilis* to acquire antimicrobial-resistant genes from other pathogens are the main causes of MDR strains (Algammal et al., 2021b).

Unfortunately, antimicrobial resistance was more common in *P. mirabilis* from dogs compared to those in human isolates (Siebor and Neuwirth, 2013). Prevalence of ampicillin resistance increased than predicted (71%) (Harada et al., 2014), which was most likely caused by TEM penicillinases (Hordijk et al., 2013). Resistance to trimethoprim/sulfamethoxazole, quinolones, and aminoglycosides to *P*. *mirabilis* restricts the therapeutic options of such antimicrobials used in the treatment of dogs infected with *P*. *mirabilis* (Wong et al., 2015).

The PCR proved that the recovered *Proteus* spp. strains from dogs are highly virulent as they harbored *ure*C, *zap*A, and *rsb*A virulence genes exhibiting prevalence as 92, 60, and 52%, respectively. The *ure*C gene causes urine pH to rise, resulting in the formation of stones (Armbruster et al., 2018). The *Proteus* spp. demonstrated in our study had 92% *ure*C amplification, indicating a higher frequency of *ure*C than other genes, thus playing a pivotal role in the virulence of *Proteus* depending on this gene. The *ure*C gene was identified in approximately 96.6% of human-infected urinary tract isolates (Ali and Yousif, 2015). In previous studies from ducks, the *ure*C gene was found in 100% of *Proteus* isolates (Algammal et al., 2021b). In addition, *Proteus* spp. lacked *rsb*A gene which encodes the characteristic swarming activity of *Proteus* and expresses a membrane sensor for promoting the extracellular polysaccharides (Walker et al., 1999; Algammal et al., 2021a). The current report showed that 15 isolates (60%) had the *zap*A gene, which codes for protease enzyme production, and *Zap*A-protease could degrade IgG, IgA1, and IgA2, controlled by the *zap*A gene (Pathirana et al., 2018; Algammal et al., 2021b).

When ESBLs are present, infectious bacteria develop resistance to lactamase, demonstrating the negative effect of the variant therapeutics (Schultz et al., 2017). ESBL-positive and -negative isolates showed different AMR rates. The mechanism of increased non-β-lactam resistance in ESBL-producing strains is unknown. ESBLs hydrolyze broad-spectrum lactam antibiotics, such as penicillins and cephalosporins in addition to piperacillin. Enterobacteriales frequently produce ESBLs. According to our results, all ESBL-positive isolates had *bla*_TEM_ gene. *P. mirabilis,* identified in the hospitalized patients’ samples, frequently contained ESBLs of the TEM type (Ahn et al., 2017; Rajivgandhi et al., 2018). The *bla*_CTX-M_ gene also exhibits resistance to cephalosporins, ceftazidime, and cefotaxime. The *bla*_SHV_ gene is commonly detected in members of the Enterobacteriaceae family (Alonso et al., 2017), which confirms our findings. Furthermore, according to our findings, the OXA-1-positive isolates showed a significantly higher prevalence than that stated in a French study (Bonnet et al., 2002). The *bla*_OXA-1_ gene promotes piperacillin and cephalosporin resistance. The *bla*_OXA-1_ and *bla*_CTX-M_ genes work together protecting *P. mirabilis* from lactam-lactamase inhibitor combinations (Schultz et al., 2017). Concurrently, *Proteus* is also resistant to sulfonamides and tetracyclines due to *sul1 and tet*A genes (Cadena et al., 2018; Algammal et al., 2021a). Class 1 integrons were connected with ESBL-producing isolates more frequently than non-ESBL-producing isolates (Chen et al., 2017). Some researchers suggest that the ESBL genes and integron are frequently connected with the *qnr* gene (Hopkins et al., 2007; Sharma et al., 2009). When bacteria are subjected to selective pressure by sulfonamides, the presence of *sul*1 genes with class 1 integrons provides a useful tool for the maintenance and subsequent development of resistance to other antimicrobial agents (Antunes et al., 2005).

Animal companions could be as reservoirs of *Proteus*, especially *P. mirabilis*, for human infection according to previous research that found human and animals infections with closely-related bacterium strains (Marques et al., 2019). *P*. *mirabilis* showed high resistance levels against the antimicrobial agents, making it a potential health threatening factor to human health due to its high elimination frequency through diarrhea in dogs.

## 5 Limitations and future recommendations

Future work is recommended to perform appropriate identification by molecular techniques like MALDI-TOF/16S rRNA gene sequencing to realize the clonal relatedness of the obtained strains.

## 6 Conclusion

Eventually, this report study could be the first study of MDR in *Proteus* spp. isolated from Egyptian dogs. *P. mirabilis* was a highly recorded prevalence in dogs, which was associated with diarrhea. The *ure*C, *zap*A, and *rsb*A virulence genes are commonly detected in the *Proteus* isolates obtained from infected dogs. The isolated *Proteus* strains showed multidrug resistance (MDR) property against penicillins, β-lactamases, sulfonamides, cephalosporins, quinolones, aminoglycosides, and tetracyclines. They often carried the *bla*_OXA-1_, *bla*_TEM_, *bla*_CTX-M_, *bla*_SHV_, *int*I1, *tet*A, *aad*A1, *qnr*A, and *sul*1 resistance genes. The potential hazards and health risks of the *Proteus* infection in dogs need more investigation. Therefore, we encourage continuous epidemiological data collection with the antimicrobial testing of susceptibility in both humans and animals and testing variant antibiotics used in human or veterinary medicinal fields, either.

## Data Availability

The original contributions presented in the study are included in the article/Supplementary Materials; further inquiries can be directed to the corresponding authors.
